# Association of Cardiovascular Risk Burden with Cardiorespiratory Fitness and Functional Performance in Patients with Post-Stroke Spasticity: A Retrospective Observational Study

**DOI:** 10.3390/jcm15176897

**Published:** 2026-09-06

**Authors:** Laura Marieta Alexa, Cristina Octaviana Daia, Alexandru Cosmin Palcau, Livia Florentina Paduraru, Alexandru Dinulescu, Daniel Alexa, Maria Magdalena Leon, Romică Sebastian Cozma, Mihai Roca, Lucia Corina Dima-Cozma

**Affiliations:** 1 Doctoral School, University of Medicine and Pharmacy “Grigore T. Popa”, 700115 Iasi, Romania; alexalaura2004@yahoo.com; 2 Neuromotor Rehabilitation Clinic, Clinical Rehabilitation Hospital, 700661 Iasi, Romania; 3 Faculty of Medicine, “Carol Davila” University of Medicine and Pharmacy, 050474 Bucharest, Romania; livia.paduraru@umfcd.ro (L.F.P.); alexandru.dinulescu@drd.umfcd.ro (A.D.); 4 Department of Physical Medicine and Rehabilitation, C.F. 2 Clinical Hospital, 011464 Bucharest, Romania; 5 Department of General Surgery, C.F. 2 Clinical Hospital, 011464 Bucharest, Romania; 6 Department of Cardiology, Elias Emergency University Hospital, 011461 Bucharest, Romania; 7 Department of Pediatrics, Emergency Hospital for Children “Grigore Alexandrescu”, 011743 Bucharest, Romania; 8 Department of Neurology, Clinical Rehabilitation Hospital, 700661 Iasi, Romania; alexadaniel2004@yahoo.com; 9 Faculty of Medicine, University of Medicine and Pharmacy “Grigore T. Popa”, 700111 Iasi, Romania; leon_mariamagdalena@yahoo.com (M.M.L.); sebastian.cozma@umfiasi.ro (R.S.C.); mihai.c.roca@umfiasi.ro (M.R.); cozma.dima@umfiasi.ro (L.C.D.-C.); 10 Department of Cardiology, Clinical Rehabilitation Hospital, 700661 Iasi, Romania; 11 Respiratory Rehabilitation Department, Clinical Rehabilitation Hospital, 700661 Iasi, Romania

**Keywords:** post-stroke spasticity, cardiovascular risk factors, cardiopulmonary exercise testing, renal function, functional outcomes, rehabilitation

## Abstract

**Background:** Post-stroke spasticity (PSS) is a frequent and disabling complication of stroke that adversely affects mobility, exercise capacity, and functional independence. Although cardiovascular comorbidities are common in this population, their independent associations with cardiorespiratory and functional outcomes remain insufficiently characterized. This study aimed to describe the cardiovascular risk profile of patients with PSS and identify factors independently associated with cardiorespiratory and functional performance. **Methods:** This retrospective observational study included 117 patients with persistent PSS evaluated at least 3 months after stroke onset. Cardiorespiratory performance was assessed using cardiopulmonary exercise testing (CPET), while functional outcomes were evaluated using the 10-m walk test (10MWT), Barthel Index (BI), and Modified Rankin Scale (mRS). Multivariable linear regression analyses were performed to identify factors independently associated with each outcome. **Results:** Cardiovascular comorbidities were highly prevalent, particularly arterial hypertension (83.8%), dyslipidemia (59.8%), diabetes mellitus (35.9%), and coronary artery disease (29.1%). Estimated glomerular filtration rate (eGFR) showed the strongest and most consistent independent relationships with the evaluated outcomes, being associated with better cardiorespiratory performance, higher BI scores, and shorter 10MWT completion time. Active smoking, elevated HbA1c, coronary artery disease, and increased C-reactive protein were independently associated with poorer cardiorespiratory and functional performance. No significant associations were observed between cardiovascular risk factors and mRS. **Conclusions:** Cardiovascular, metabolic, inflammatory, and renal factors are independently associated with cardiorespiratory and functional outcomes in patients with PSS. Renal function showed the most consistent independent associations, while poor glycemic control, active smoking, coronary artery disease, and systemic inflammation were also independently associated with poorer cardiorespiratory and functional outcomes. These findings support systematic cardiovascular risk assessment to facilitate individualized risk stratification and optimize post-stroke rehabilitation.

## 1. Introduction

Stroke remains one of the most pressing public health challenges of the twenty-first century. According to the World Stroke Organization Global Stroke Fact Sheet 2025, approximately 12 million people experience a stroke each year worldwide, with more than 7 million deaths annually attributed to this condition. Between 1990 and 2021, the global burden of stroke increased substantially, with a 70% rise in incident strokes, an 86% increase in prevalent cases, and a 44% increase in stroke-related deaths, most of this burden occurring in low- and middle-income countries [1]. Global projections further suggest that, in the absence of effective preventive strategies, the number of stroke survivors may reach 159 million by 2050 [2,3].

Beyond its immediate neurological consequences, stroke is characterized by a complex interplay of cardiovascular risk factors that not only predispose individuals to the initial cerebrovascular event but also influence the trajectory of functional recovery. Hypertension, diabetes mellitus, dyslipidemia, coronary artery disease, smoking, and renal dysfunction represent the principal modifiable risk factors associated with both stroke incidence and recurrence [4,5,6]. Metabolic risk factors alone account for approximately 69% of all strokes worldwide, underscoring the central role of cardiovascular multimorbidity in determining post-stroke outcomes [1].

Post-stroke spasticity (PSS) is among the most clinically significant sequelae of stroke, affecting an estimated 25–43% of survivors within weeks to months after the acute event [7]. PSS is characterized by a velocity-dependent increase in muscle tone resulting from upper motor neuron injury, leading to impaired upper-limb function, reduced mobility, and limitations in activities of daily living [8,9]. Persistent spasticity may subsequently result in joint contractures, pressure ulcers, and chronic pain, contributing to a fourfold increase in caregiver burden [10]. From a pathophysiological perspective, PSS arises from disrupted excitatory and inhibitory signaling within descending motor pathways and spinal circuits secondary to cortical and subcortical ischemic injury. These alterations lead to disinhibition of spinal reflex pathways and abnormal motor control [11,12].

Another important consequence of stroke that has received increasing attention is the marked reduction in cardiorespiratory fitness observed among survivors [13,14]. Peak oxygen uptake (VO_2_peak) is considered the gold-standard measure of cardiorespiratory fitness [15]. VO_2_peak has been reported to decrease by nearly 50% during the first week after stroke compared with age-matched healthy individuals. Consequently, stroke survivors require a higher aerobic capacity than healthy peers to perform routine daily activities because of the biomechanical inefficiency associated with hemiparesis and spasticity [16,17]. This paradox creates a state of physiological vulnerability that limits rehabilitation potential and functional independence. A recent systematic review further confirmed that stroke survivors exhibit substantially reduced exercise capacity compared with age-matched healthy individuals, with important implications for activities of daily living and long-term prognosis [18].

This interaction may be particularly relevant in patients with persistent PSS. Spasticity occurs within a broader pattern of post-stroke motor impairment that may include weakness, abnormal muscle activation, impaired motor control, and altered gait mechanics, further reducing movement efficiency. Consequently, limited cardiorespiratory reserve and coexisting cardiovascular and metabolic comorbidities may further constrain exercise tolerance, mobility, and independence in daily activities. Patients with persistent PSS therefore represent a clinically relevant population in which to investigate the relationship between cardiovascular burden, cardiorespiratory fitness, and functional performance.

The relationship between cardiovascular and metabolic comorbidity and post-stroke recovery is complex and multidimensional. In the present study, this relationship is examined specifically in relation to cardiorespiratory fitness and functional performance, encompassing mobility, independence in activities of daily living, and global disability.

Estimated glomerular filtration rate (eGFR), a widely accepted marker of renal function, has emerged as an independent predictor of both functional outcomes and mortality in stroke populations [19]. Reduced eGFR has consistently been associated with poorer outcomes following acute stroke, while lower admission eGFR values have been linked to an increased risk of mortality [19,20]. In parallel, accumulating evidence from the general population demonstrates that impaired renal function is independently associated with poorer physical performance across multiple domains, including walking capacity, muscle strength, and overall functional status [21]. Similarly, systemic inflammation, reflected by elevated C-reactive protein (CRP) levels, has been identified as an important predictor of adverse outcomes after ischemic stroke. Increased CRP concentrations are associated with greater stroke severity, higher long-term mortality, and poorer functional recovery, as assessed by the modified Rankin Scale (mRS) [22].

Diabetes mellitus and poor glycemic control represent additional determinants of cardiorespiratory fitness. Chronic hyperglycemia promotes the formation of advanced glycation end-products, endothelial dysfunction, skeletal muscle mitochondrial impairment, and diastolic dysfunction, all of which contribute to reduced VO_2_peak and impaired exercise tolerance [23]. Furthermore, smoking adversely affects cardiorespiratory fitness and functional capacity through multiple mechanisms, including systemic inflammation, endothelial dysfunction, and vasoconstriction [24]. Together, these mechanisms further exacerbate the physiological deconditioning commonly observed in stroke survivors.

In the present study, functional performance refers to three clinically relevant domains of post-stroke functioning: mobility, independence in activities of daily living, and global disability. These domains were operationalized using the 10-Meter Walk Test, the Barthel Index, and the modified Rankin Scale, respectively. Cardiorespiratory fitness and exercise capacity were assessed separately using cardiopulmonary exercise testing.

Despite substantial advances in understanding cardiovascular risk factors after stroke, few studies have comprehensively investigated the independent associations of renal function, glycemic control, systemic inflammation, coronary artery disease, and smoking with objectively assessed cardiorespiratory fitness and functional performance specifically in patients with post-stroke spasticity. Therefore, the present study aimed to comprehensively characterize the cardiovascular risk profile of patients with post-stroke spasticity and to determine the independent associations of individual cardiovascular and metabolic risk factors with cardiorespiratory fitness and functional performance. We hypothesized that modifiable cardiovascular risk factors would be independently associated with exercise capacity and functional outcomes in this population.

## 2. Materials and Methods

### 2.1. Study Design and Patient Population

This single-center retrospective observational study included consecutive patients with post-stroke spasticity who were evaluated at the Neuromotor Rehabilitation Clinic of the Clinical Rehabilitation Hospital, Iași, Romania. Patient data were collected between January 2024 and August 2025. Statistical analyses were subsequently performed between December 2025 and March 2026.

Patients were eligible for inclusion if they met all of the following criteria: age ≥ 18 years; a confirmed diagnosis of ischemic stroke; persistent post-stroke spasticity assessed at least 3 months after stroke onset; hemodynamic stability at the time of assessment; the ability to perform cardiopulmonary exercise testing (CPET); and the absence of pain severe enough to interfere with exercise testing.

The cohort was restricted to patients with ischemic stroke to reduce etiological and pathophysiological heterogeneity, as ischemic and hemorrhagic stroke differ in underlying mechanisms, clinical characteristics, and recovery trajectories. This restriction was intended to improve the interpretability of the associations between cardiovascular and metabolic factors, cardiorespiratory fitness, and functional performance.

Post-stroke spasticity was diagnosed by a specialist in Physical and Rehabilitation Medicine and was defined as a velocity-dependent increase in muscle tone attributable to the index stroke. The diagnosis was established during neurological examination based on increased resistance to passive movement. Spasticity severity was assessed as part of the routine clinical evaluation using the Modified Ashworth Scale (MAS) for relevant muscle groups of the affected upper and lower limbs and was documented in the medical records. However, individual MAS scores were not extracted into the study database because spasticity severity was not predefined as an analytical variable in the original study design. Consequently, MAS severity was not included in the statistical analyses. None of the participants were receiving specific treatment for spasticity at the time of the study assessments. The study population consisted of ambulatory patients with clinically mild-to-moderate spasticity that did not preclude walking and allowed participation in the functional and exercise assessments. No oral antispastic medication, botulinum toxin treatment, or other targeted antispastic intervention was being administered.

Patients were excluded if they had neurological disorders other than stroke that could interfere with the interpretation of functional or cardiorespiratory assessments. Additional exclusion criteria included orthopedic conditions precluding or substantially limiting exercise testing (e.g., fractures or severe hip or knee osteoarthritis), untreated psychotic or neurotic disorders that could impair the patient’s ability to understand instructions, cooperate with the assessments, or safely perform CPET, uncontrolled hypertension, cardiac arrhythmias or other unstable cardiovascular conditions, recent pulmonary embolism, subacute systemic illness, active infection, or any other contraindication to CPET. Stable and adequately treated psychiatric conditions that did not interfere with the patient’s ability to understand instructions or participate in the assessments were not considered exclusion criteria.

### 2.2. Clinical and Laboratory Assessments

Demographic and anthropometric characteristics collected for each participant included age, sex, body mass index (BMI), and waist circumference. Cardiovascular risk factors evaluated included arterial hypertension, diabetes mellitus, dyslipidemia, coronary artery disease (CAD), and smoking status, categorized as never-smoker, former smoker, or current smoker. Arterial hypertension, diabetes mellitus, dyslipidemia, and coronary artery disease were considered present when a previously established diagnosis by the relevant treating specialist was documented in the patient’s medical records. These diagnoses were therefore based on the documented medical history and were not established de novo from laboratory values or clinical measurements obtained during the study admission.

The laboratory parameters evaluated were glycated hemoglobin (HbA1c), low-density lipoprotein (LDL) cholesterol, hemoglobin (Hb), estimated glomerular filtration rate (eGFR), and C-reactive protein (CRP). Laboratory values were obtained during the same rehabilitation admission and extracted from the patients’ medical records. Estimated glomerular filtration rate was automatically calculated by the hospital laboratory. HbA1c and LDL cholesterol values obtained during the admission were analyzed as laboratory variables and were not used to retrospectively redefine the pre-existing diagnoses of diabetes mellitus or dyslipidemia.

Stroke-related variables included lesion laterality (right-sided, left-sided, or bilateral/other) and time since stroke onset. For group comparisons and multivariable analyses, smoking status was analyzed as a binary variable (current smokers versus non-smokers), with the non-smoker category including both never-smokers and former smokers.

### 2.3. Cardiopulmonary Exercise Testing

Cardiorespiratory fitness was assessed using cardiopulmonary exercise testing (CPET), performed on an electronically braked cycle ergometer with a progressive incremental protocol. The primary CPET outcome was peak oxygen uptake (VO_2_peak), expressed as mL/kg/min. VO_2_peak was defined as the highest oxygen consumption achieved during the final 20–30 s of symptom-limited incremental exercise.

Secondary CPET outcomes included the anaerobic threshold (AT), exercise duration, and maximum workload achieved. The anaerobic threshold was defined as the point during incremental exercise at which anaerobic metabolism begins to supplement aerobic metabolism. AT was determined using a combined approach consisting of the V-slope method (the point of disproportionate increase in carbon dioxide production [VCO_2_] relative to oxygen uptake [VO_2_]) and the ventilatory equivalents method (an increase in VE/VO_2_ without a concurrent increase in VE/VCO_2_). Both criteria were automatically identified by the BTL CardioPoint software and subsequently reviewed and confirmed by the supervising physician.

CPET was performed using BTL CardioPoint software (version 2.32; BTL Industries, Prague, Czech Republic) connected to an electronically braked cycle ergometer (Ergoline Ergoselect 100, Bitz, Baden-Württemberg, Germany). Following a resting baseline period, exercise began at an initial workload of 10 W with a continuous ramp increment of 15 W/min, resulting in a typical exercise duration of 8–12 min. A 10-min recovery period was monitored after exercise.

During CPET, breath-by-breath respiratory gas exchange was continuously recorded to determine peak oxygen uptake (VO_2_peak), anaerobic threshold (AT), exercise duration, and maximum workload achieved. Continuous 12-lead electrocardiographic monitoring was performed throughout the test. Blood pressure was measured every two minutes using the manual auscultatory method. Perceived exertion at peak exercise was assessed using the 6–20 Borg Rating of Perceived Exertion scale.

Testing was terminated if any of the following occurred: onset of angina or ischemic ST-segment changes on the electrocardiogram; a decrease in systolic blood pressure of ≥10 mmHg despite increasing workload; severe hypertension (systolic blood pressure > 250 mmHg or diastolic blood pressure > 115 mmHg); signs of poor peripheral perfusion (e.g., pallor or cyanosis); moderate-to-severe angina, dyspnea, or dizziness; new-onset clinically significant ventricular or supraventricular arrhythmias; technical difficulties preventing adequate patient monitoring; or the patient’s request to stop the test.

### 2.4. Functional Assessments

Functional performance was assessed across three domains: mobility, independence in activities of daily living, and global disability. These domains were evaluated using the 10-Meter Walk Test (10MWT), the Barthel Index (BI), and the modified Rankin Scale (mRS), respectively. All assessments were administered on the same day as the cardiopulmonary exercise test by trained members of the rehabilitation team.

Walking performance was assessed using the 10MWT, a validated measure of walking performance in individuals with stroke [25]. Participants walked a marked 10-m distance at their comfortable self-selected pace, starting from a standing position. The use of a customary walking aid (e.g., a cane or walker) was permitted when required for safety. The outcome recorded was the time required to complete the 10-m distance, expressed in seconds. A single timed trial was used because the study retrospectively analyzed routinely collected clinical data, and the local functional assessment protocol recorded one timed 10MWT trial per patient rather than repeated trials. Consequently, a mean of multiple trials was not available for retrospective analysis.

Activities of daily living were assessed using the BI, a widely established measure of functional independence in stroke, scored from 0 to 100, with higher scores indicating greater functional independence [26]. The BI was completed by the treating physician or physiotherapist based on direct clinical observation supplemented by patient or caregiver interview.

Global disability was assessed using the mRS, a widely used and validated measure of global disability after stroke, scored from 0 to 6, with higher scores indicating greater disability [27,28]. The mRS was independently assessed by two trained raters, and any discrepancies were resolved by consensus.

### 2.5. Statistical Analysis

Statistical analyses were performed using IBM SPSS Statistics version 25 (IBM Corp., Armonk, NY, USA). Continuous variables were tested for normality using the Shapiro–Wilk test. Normally distributed variables are presented as mean ± standard deviation (SD), whereas non-normally distributed variables are presented as median and interquartile range (IQR). Categorical variables are presented as frequencies and percentages.

Because of the retrospective study design, no formal a priori sample size calculation was performed. Nevertheless, the final study population comprised 117 participants, providing approximately 23 observations per predictor in the largest multivariable model, which exceeds commonly recommended thresholds for linear regression.

Comparisons between two independent groups were performed using the independent-samples *t*-test for normally distributed variables and the Mann–Whitney U test for non-normally distributed variables. Homogeneity of variances was assessed using Levene’s test, and the appropriate *t*-test results (equal or unequal variances assumed) were reported accordingly. Associations between two continuous normally distributed variables were evaluated using Pearson’s correlation coefficient, whereas associations involving at least one non-normally distributed variable were assessed using Spearman’s rank correlation coefficient. These univariate analyses were retained to provide a transparent description of the crude relationships between individual clinical variables and the evaluated outcomes, whereas the subsequent multivariable regression models were used to assess adjusted associations.

Factors independently associated with cardiorespiratory fitness and functional performance were evaluated using multivariable linear regression analyses with the enter method. Candidate variables were considered on the basis of their established or biologically plausible relationships with cardiovascular health, cardiorespiratory fitness, and post-stroke functional performance, as supported by previous literature [29]. These included cardiovascular and metabolic comorbidities, smoking, anthropometric factors, renal function, glycemic control, systemic inflammation, and other clinically relevant characteristics available in the retrospective dataset. The final set of covariates entered into each outcome-specific model was restricted to maintain parsimonious models and an observations-to-predictor ratio greater than 20:1, thereby reducing the risk of overfitting. Regression results are presented as unstandardized coefficients (B), standardized coefficients (β), 95% confidence intervals (95% CIs), and *p*-values. Multicollinearity was assessed using variance inflation factors (VIFs) and tolerance statistics. Statistical significance was defined as *p* < 0.05.

All regression assumptions were verified before model interpretation. Tolerance values were >0.20 and VIF values were <2, indicating the absence of multicollinearity. Visual inspection of residual plots demonstrated acceptable linearity and homoscedasticity, while normal probability plots indicated that residuals were approximately normally distributed. Durbin–Watson statistics ranged from 1.86 to 2.15, confirming the independence of residuals. Across all multivariable models, the observations-to-predictor ratio ranged from 23.4 to 39, exceeding commonly recommended thresholds for linear regression. The differences between R^2^ and adjusted R^2^ were consistently small (0.020–0.032), indicating good model stability and a low risk of overfitting. Cohen’s effect sizes ranged from moderate to very large (f^2^ = 0.27–1.01), corresponding to high or excellent statistical power across all models. No missing data were identified; therefore, complete-case analyses were performed.

### 2.6. Ethical Considerations

The study protocol was approved by the Ethics Committee of the “Grigore T. Popa” University of Medicine and Pharmacy, Iași, Romania (Approval No. 321/08.06.2023), and by the Ethics Committee of the Clinical Rehabilitation Hospital, Iași, Romania (Approval No. 01/10.02.2023). All procedures were conducted in accordance with the ethical principles of the Declaration of Helsinki. Written informed consent was obtained from all patients at the time of hospital admission as part of the standard institutional procedure and included consent for the subsequent use of clinical and medical record data for research purposes. The retrospective use of these data for the present doctoral research project was subsequently approved by the institutional ethics committees.

## 3. Results

### 3.1. Main Characteristics of the Population

A total of 117 patients with post-stroke spasticity were included in the study. The study population was balanced by sex, comprising 59 men (50.4%) and 58 women (49.6%), with a mean age of 74.3 ± 7.9 years. The mean body mass index (BMI) was 27.8 ± 4.5 kg/m^2^, and the mean waist circumference was 103.5 ± 15.1 cm. Hypertension was the most prevalent cardiovascular risk factor (83.8%), followed by dyslipidemia (59.8%), a history of smoking (48.7%), diabetes mellitus (35.9%), and coronary artery disease (29.1%). Median HbA1c was 6.0% (IQR, 5.7–7.7), mean LDL cholesterol was 102.3 ± 23.8 mg/dL, mean estimated glomerular filtration rate (eGFR) was 68.3 ± 16.3 mL/min/1.73 m^2^, mean hemoglobin was 13.3 ± 1.1 g/dL, and median C-reactive protein (CRP) was 3.8 mg/L (IQR, 1.95–5.55). Right-sided stroke was the most frequent lesion location (54.7%), followed by left-sided stroke (41.9%). The median time since stroke was 11 months (IQR, 7–20). Baseline demographic, clinical, anthropometric, and laboratory characteristics of the study population are summarized in Table 1.

### 3.2. Peak Oxygen Uptake

The mean peak oxygen uptake (VO_2_peak) was 12.53 ± 1.44 mL/kg/min. Patients with diabetes mellitus had significantly lower VO_2_peak values than those without diabetes (12.1 ± 1.4 vs. 12.9 ± 1.3 mL/kg/min, *p* = 0.003). Higher HbA1c levels were associated with lower VO_2_peak (ρ = −0.210, *p* = 0.023). Similarly, patients with coronary artery disease had significantly lower VO_2_peak than those without CAD (12.01 ± 1.36 vs. 12.75 ± 1.43 mL/kg/min, *p* = 0.011). Estimated glomerular filtration rate (eGFR) was positively correlated with VO_2_peak (r = 0.495, *p* < 0.001), whereas CRP was inversely correlated with VO_2_peak (ρ = −0.221, *p* = 0.017). Current smokers also had significantly lower VO_2_peak than non-smokers (11.73 ± 1.42 vs. 12.70 ± 1.39 mL/kg/min, *p* = 0.005). No significant associations were observed for sex, age, BMI, waist circumference, hypertension, dyslipidemia, LDL cholesterol, hemoglobin, stroke location, or time since stroke.

Multivariable linear regression showed that eGFR had the strongest independent positive association with VO_2_peak (β = 0.508, *p* < 0.001), whereas HbA1c (β = −0.229, *p* = 0.001), coronary artery disease (β = −0.304, *p* < 0.001), current smoking (β = −0.297, *p* < 0.001), and CRP (β = −0.183, *p* = 0.010) were independently associated with lower VO_2_peak values. The regression model explained 50.2% of the variance in VO_2_peak (R^2^ = 0.502; adjusted R^2^ = 0.479; F (5,111) = 22.335, *p* < 0.001), and all regression assumptions were satisfied (Table 2).

### 3.3. Anaerobic Threshold

Mean anaerobic threshold (AT) was 10.11 ± 1.58 mL/kg/min. Patients with diabetes mellitus had significantly lower AT values than those without diabetes (9.60 ± 1.55 vs. 10.39 ± 1.54 mL/kg/min, *p* = 0.009). Higher HbA1c levels were associated with lower AT (ρ = −0.216, *p* = 0.019). Similarly, patients with coronary artery disease had significantly lower AT than those without CAD (9.57 ± 1.43 vs. 10.33 ± 1.60 mL/kg/min, *p* = 0.019). Estimated glomerular filtration rate (eGFR) was positively correlated with AT (r = 0.437, *p* < 0.001), whereas current smokers had significantly lower AT than non-smokers (9.18 ± 1.41 vs. 10.30 ± 1.55 mL/kg/min, *p* = 0.004). No significant associations were observed for sex, age, BMI, waist circumference, hypertension, dyslipidemia, LDL cholesterol, hemoglobin, C-reactive protein, stroke location, or time since stroke.

Multivariable linear regression showed that eGFR had the strongest independent positive association with AT (β = 0.441, *p* < 0.001), whereas HbA1c (β = −0.221, *p* = 0.004), coronary artery disease (β = −0.262, *p* = 0.001), and current smoking (β = −0.324, *p* < 0.001) were independently associated with lower AT values. The regression model explained 39.5% of the variance in AT (R^2^ = 0.395; adjusted R^2^ = 0.374; F (4,112) = 18.294, *p* < 0.001), and all regression assumptions were satisfied (Table 3).

### 3.4. Exercise Duration

Median exercise duration was 7.3 min (IQR, 6.9–8.0). Patients with hypertension had shorter exercise duration than those without hypertension (7.2 [IQR, 6.8–7.92] vs. 7.7 [IQR, 7.4–8.1] min, *p* = 0.038). Higher CRP levels were associated with shorter exercise duration (ρ = −0.326, *p* < 0.001), whereas estimated glomerular filtration rate (eGFR) was positively correlated with exercise duration (ρ = 0.426, *p* < 0.001). Patients with diabetes mellitus also had shorter exercise duration than those without diabetes (7.1 [IQR, 6.7–7.52] vs. 7.6 [IQR, 7.0–8.0] min, *p* = 0.026). Current smokers had significantly shorter exercise duration than non-smokers (7.05 [IQR, 6.6–7.47] vs. 7.4 [IQR, 6.95–8.0] min, *p* = 0.027). No significant associations were observed for sex, age, BMI, waist circumference, dyslipidemia, LDL cholesterol, hemoglobin, HbA1c, coronary artery disease, stroke location, or time since stroke.

Multivariable linear regression showed that eGFR had the strongest independent positive association with exercise duration (β = 0.434, *p* < 0.001), whereas current smoking (β = −0.235, *p* = 0.004), CRP (β = −0.188, *p* = 0.021), and hypertension (β = −0.162, *p* = 0.042) were independently associated with shorter exercise duration. Diabetes mellitus was no longer independently associated with exercise duration after adjustment for the other covariates (*p* = 0.155). The regression model explained 29.1% of the variance in exercise duration (R^2^ = 0.291; adjusted R^2^ = 0.259; F (5,111) = 9.093, *p* < 0.001), and all regression assumptions were satisfied (Table 4).

### 3.5. Maximum Workload

Mean maximum workload was 49.42 ± 7.51 W. Patients with diabetes mellitus achieved a significantly lower maximum workload than those without diabetes (47.67 ± 7.53 vs. 50.40 ± 7.37 W, *p* = 0.032). Estimated glomerular filtration rate (eGFR) was positively correlated with maximum workload (r = 0.400, *p* < 0.001), whereas body mass index (BMI) was inversely correlated (r = −0.219, *p* = 0.018). Current smokers also demonstrated lower maximum workload than non-smokers (48.05 ± 5.66 vs. 50.70 ± 7.84 W, *p* = 0.019). No significant associations were observed for sex, age, waist circumference, hypertension, dyslipidemia, LDL cholesterol, HbA1c, coronary artery disease, hemoglobin, C-reactive protein, stroke location, or time since stroke.

Multivariable linear regression showed that eGFR was independently associated with higher maximum workload (β = 0.403, *p* < 0.001), whereas higher BMI was independently associated with lower maximum workload (β = −0.224, *p* = 0.007). Current smoking and diabetes mellitus were not statistically significant after adjustment for the other covariates (Table 5).

### 3.6. 10-Meter Walk Test Completion Time

Mean 10MWT completion time was 17.54 ± 1.93 s. Higher C-reactive protein (CRP) levels were associated with longer 10MWT completion time (ρ = 0.255, *p* = 0.006), whereas estimated glomerular filtration rate (eGFR) was inversely correlated with 10MWT completion time (r = −0.241, *p* = 0.009). Current smokers demonstrated significantly longer 10MWT completion time than non-smokers (18.45 ± 2.00 vs. 17.35 ± 1.88 s, *p* = 0.021). Similarly, patients with coronary artery disease had significantly longer 10MWT completion times than those without coronary artery disease (18.15 ± 2.02 vs. 17.28 ± 1.85 s, *p* = 0.027). No significant associations were observed for sex, age, diabetes mellitus, HbA1c, BMI, waist circumference, hypertension, dyslipidemia, LDL cholesterol, hemoglobin, stroke location, or time since stroke.

Multivariable linear regression showed that higher eGFR was independently associated with shorter 10MWT completion time (β = −0.278, *p* = 0.001), whereas current smoking (β = 0.235, *p* = 0.007), higher CRP levels (β = 0.222, *p* = 0.011), and coronary artery disease (β = 0.254, *p* = 0.003) were independently associated with longer 10MWT completion times. The regression model explained 21.3% of the variance in 10-m walk time (R^2^ = 0.213; adjusted R^2^ = 0.185; F (4,112) = 7.571, *p* < 0.001), and all regression assumptions were satisfied (Table 6).

### 3.7. Barthel Index

Mean Barthel Index score was 70.03 ± 8.18. Estimated glomerular filtration rate (eGFR) was positively correlated with the Barthel Index (r = 0.378, *p* < 0.001). Current smokers had significantly lower Barthel Index scores than non-smokers (65.3 ± 9.40 vs. 71.00 ± 7.59, *p* = 0.004). Similarly, patients with diabetes mellitus (65.57 ± 8.66 vs. 71.40 ± 7.61, *p* = 0.015), coronary artery disease (66.97 ± 7.12 vs. 71.28 ± 8.29, *p* = 0.009), and dyslipidemia (68.61 ± 7.68 vs. 72.13 ± 8.52, *p* = 0.022) had significantly lower Barthel Index scores than those without these conditions. No significant associations were observed for sex, age, HbA1c, BMI, waist circumference, hypertension, LDL cholesterol, hemoglobin, C-reactive protein, stroke location, or time since stroke.

Multivariable linear regression showed that eGFR had the strongest independent positive association with Barthel Index score (β = 0.408, *p* < 0.001), whereas current smoking (β = −0.329, *p* < 0.001), coronary artery disease (β = −0.268, *p* = 0.001), dyslipidemia (β = −0.199, *p* = 0.011), and diabetes mellitus (β = −0.155, *p* = 0.050) were independently associated with lower Barthel Index scores. The regression model explained 38.2% of the variance in Barthel Index (R^2^ = 0.382; adjusted R^2^ = 0.355; F (5,111) = 13.742, *p* < 0.001), and all regression assumptions were satisfied (Table 7).

### 3.8. Modified Rankin Scale (mRS)

The modified Rankin Scale (mRS) showed a median score of 2 (IQR, 2–3). No significant associations were observed for sex, age, body mass index, waist circumference, hypertension, diabetes mellitus, HbA1c, dyslipidemia, LDL cholesterol, coronary artery disease, estimated glomerular filtration rate, hemoglobin, C-reactive protein, current smoking, stroke location, or time since stroke. Because no variables reached statistical significance in the univariate analyses and the modified Rankin Scale is an ordinal outcome with limited score variability, multivariable regression analysis was not considered appropriate.

## 4. Discussion

### 4.1. Principal Findings in Context

The present study investigated the cardiovascular risk profile, cardiorespiratory fitness, and functional performance of 117 patients with post-stroke spasticity. Several cardiovascular, metabolic, inflammatory, and lifestyle-related factors were associated with reduced exercise capacity and poorer functional outcomes, although their independent contributions differed across the assessed variables. Renal function emerged as the only factor consistently and independently associated with all evaluated cardiorespiratory and functional outcomes. Higher eGFR was associated with greater peak oxygen uptake, anaerobic threshold, exercise duration, maximum workload, and Barthel Index scores, as well as with shorter 10MWT completion time. This association is clinically relevant in the context of post-stroke spasticity and is biologically plausible given the close relationships among renal function, cardiovascular health, systemic metabolism, and skeletal muscle physiology [30].

The mean VO_2_peak observed in our cohort was 12.53 ± 1.44 mL/kg/min, supporting the presence of markedly reduced cardiorespiratory fitness in patients with post-stroke spasticity. Previous studies have consistently demonstrated substantial impairment in aerobic capacity after stroke, with meta-analytic data indicating that VO_2_peak may decline by approximately 50% during the first week after the acute event [17]. In addition, hemiparesis, impaired motor control, and reduced movement efficiency increase the relative aerobic cost of routine activities, causing stroke survivors to use a greater proportion of their available cardiorespiratory reserve during daily tasks [16]. The low VO_2_peak observed at a median of 11 months after stroke indicates that impaired aerobic capacity persists into the chronic rehabilitation phase, when neurological deficits, physical deconditioning, spasticity, and associated comorbidities may continue to restrict exercise tolerance and functional recovery. In patients with persistent PSS, these relationships should also be interpreted within the broader context of post-stroke motor impairment. Spasticity commonly coexists with muscle weakness, abnormal muscle activation, impaired motor control, and altered movement patterns, all of which may reduce movement efficiency and increase the physiological demands of walking and other functional activities. Consequently, reduced cardiorespiratory reserve and coexisting cardiovascular or metabolic comorbidities may become particularly relevant when superimposed on an already inefficient motor system. The associations observed in the present study may therefore reflect the combined influence of systemic cardiovascular and metabolic burden and stroke-related motor impairment on exercise tolerance and functional performance. However, because detailed measures of motor impairment and individual MAS scores were not included in the analytical dataset, these potential interactions could not be directly examined and should be considered hypothesis-generating.

### 4.2. The Role of Renal Function in Cardiorespiratory and Functional Performance

Renal function emerged as the only factor consistently and independently associated with all evaluated cardiorespiratory and functional outcomes. Higher eGFR was independently associated with VO_2_peak (β = 0.508), anaerobic threshold (β = 0.441), exercise duration (β = 0.434), maximum workload (β = 0.403), and Barthel Index score (β = 0.408), whereas it was associated with shorter 10MWT completion time (β = −0.278). Collectively, these findings indicate that renal function may represent an important systemic correlate of rehabilitation performance in patients with post-stroke spasticity.

This observation is consistent with evidence from non-stroke populations. In a large cross-sectional analysis of 7396 participants from the Maastricht Study, eGFR values below 60 mL/min/1.73 m^2^ were associated with shorter six-minute walk distance, poorer timed chair-rise performance, and reduced grip and elbow-flexion strength [21]. In patients with acute stroke, reduced eGFR has also been independently associated with poorer functional outcomes and increased mortality, with values below 48.5 mL/min/1.73 m^2^ demonstrating discriminatory value for mortality risk [19].

The mean eGFR in our cohort was 68.33 ± 16.26 mL/min/1.73 m^2^, corresponding numerically to the mildly reduced G2 filtration range. However, this value alone does not establish a diagnosis of chronic kidney disease, which requires persistent renal impairment and/or additional markers of kidney damage. Nevertheless, the consistency of the associations observed in the present study suggests that even modest reductions in renal filtration may be clinically relevant to exercise capacity and functional performance after stroke.

Several mechanisms may contribute to this relationship, including endothelial dysfunction, systemic inflammation, accumulation of uremic toxins, mitochondrial abnormalities, impaired muscle protein metabolism, and skeletal muscle wasting. These mechanisms may reduce oxygen delivery and utilization, impair muscle strength and endurance, and aggravate physical deconditioning. Anemia may additionally contribute in patients with more advanced renal impairment, although hemoglobin was not associated with the evaluated outcomes in the present cohort.

### 4.3. Diabetes Mellitus and Glycemic Control

In the univariate analyses, diabetes mellitus was associated with lower VO_2_peak, lower anaerobic threshold, shorter exercise duration, lower maximum workload, and lower Barthel Index scores. After multivariable adjustment, HbA1c remained independently associated with VO_2_peak and anaerobic threshold, whereas diabetes mellitus showed only a borderline independent association with the Barthel Index. Higher HbA1c was independently associated with lower VO_2_peak (β = −0.229) and lower anaerobic threshold (β = −0.221), indicating that poorer glycemic control was related to reduced aerobic performance. Chronic hyperglycemia may impair cardiorespiratory function through endothelial dysfunction, reduced vascular reactivity, altered myocardial relaxation, microvascular injury, mitochondrial dysfunction, and impaired peripheral oxygen extraction [23,31]. The inverse correlation between HbA1c and VO_2_peak observed in the present study is consistent with findings from cardiac rehabilitation populations, in which poorer glycemic control has been associated with attenuated improvements in peak oxygen uptake during structured exercise programs [32].

These results support the integration of glycemic assessment and optimization into comprehensive post-stroke rehabilitation. Glycemic control remains essential for secondary vascular prevention and may also be relevant to the preservation of aerobic capacity and functional independence. However, because of the observational design of the present study, these findings should be interpreted as associations rather than evidence of a direct causal effect of glycemic optimization on rehabilitation outcomes.

### 4.4. Coronary Artery Disease

Coronary artery disease was independently associated with lower VO_2_peak (β = −0.304), lower anaerobic threshold (β = −0.262), longer 10MWT completion time (β = 0.254), and lower Barthel Index scores (β = −0.268). These associations persisted after adjustment for the other variables included in the respective models. The presence of coronary artery disease may limit exercise performance through reduced myocardial perfusion, impaired chronotropic response, lower cardiac reserve, and an increased likelihood of exercise-related symptoms or early fatigue [33]. In patients with stroke, these cardiovascular limitations may compound the reduced movement efficiency and increased energetic cost caused by hemiparesis and spasticity.

Previous studies have shown that rehabilitation programs incorporating principles of cardiac rehabilitation may improve cardiovascular performance and overall function in stroke survivors and may be associated with lower long-term mortality [34]. The present findings therefore support careful cardiovascular assessment and individualized exercise prescription in patients with post-stroke spasticity, particularly in those with coexisting coronary artery disease.

### 4.5. Systemic Inflammation

Higher C-reactive protein levels were independently associated with lower VO_2_peak (β = −0.183), shorter exercise duration (β = −0.188), and longer 10MWT completion time (β = 0.222). These findings are consistent with evidence linking systemic inflammation to impaired recovery and poorer functional outcomes after stroke [35,36,37]. Previous studies have associated elevated CRP concentrations with greater post-stroke disability, poorer functional recovery, and less favorable outcomes after acute treatment [22,38]. Systemic inflammation may impair skeletal muscle oxidative metabolism, promote protein catabolism, contribute to endothelial dysfunction, and exacerbate physical deconditioning [39]. These effects may be especially relevant in patients with post-stroke spasticity, in whom reduced mobility and abnormal muscle activation may already compromise muscle metabolism and exercise efficiency.

The lack of an independent association between CRP and the Barthel Index or mRS suggests that associations with systemic inflammation may be more readily detected through quantitative measures of aerobic capacity and walking performance than through broader measures of disability.

### 4.6. Current Smoking

Current smoking was independently associated with lower VO_2_peak (β = −0.297), lower anaerobic threshold (β = −0.324), shorter exercise duration (β = −0.235), lower Barthel Index scores (β = −0.329), and longer 10MWT completion time (β = 0.235). Current smokers also demonstrated lower maximum workload in the univariate analysis, although this association was no longer significant after multivariable adjustment. The relatively large standardized coefficients observed for current smoking in the anaerobic threshold and Barthel Index models indicate a substantial association with both cardiorespiratory performance and functional independence. Smoking may impair rehabilitation performance through endothelial dysfunction, reduced oxygen delivery, increased carboxyhemoglobin levels, systemic inflammation, impaired pulmonary function, and accelerated atherosclerotic disease [40]. In stroke survivors, these effects may further reduce the physiological reserve available to compensate for neurological impairment.

Exercise-based rehabilitation can improve cardiorespiratory fitness and walking performance after stroke [41]. Smoking cessation should therefore be integrated into secondary prevention and rehabilitation programs to reduce cardiovascular risk and potentially improve the physiological conditions required for functional recovery.

### 4.7. Additional Cardiovascular and Metabolic Risk Factors

Higher BMI was independently associated only with lower maximum workload (β = −0.224). This finding may reflect the greater mechanical and metabolic demands imposed by excess body weight during physical effort. The absence of independent associations with VO_2_peak, anaerobic threshold, exercise duration, walking performance, or functional independence suggests that BMI did not exert a uniform influence across all outcomes.

Hypertension showed a modest independent association with shorter exercise duration (β = −0.162). This result suggests that the cumulative cardiovascular burden associated with hypertension may contribute to reduced submaximal exercise tolerance. However, given the high prevalence of hypertension in the cohort and the relatively small standardized coefficient, this association should be interpreted cautiously.

Dyslipidemia was independently associated with lower Barthel Index scores (β = −0.199), although it was not independently associated with the cardiorespiratory outcomes. This pattern may indicate that dyslipidemia is more closely related to cumulative vascular burden and global functional status than to exercise physiology itself. The absence of an association between LDL cholesterol and the evaluated outcomes may reflect lipid-lowering treatment, limited variability in LDL values, or residual confounding. Therefore, dyslipidemia should be interpreted as a broader vascular risk marker rather than as evidence of a direct effect of circulating LDL cholesterol on functional performance.

Age should also be considered when interpreting these findings. The study population was predominantly older, with a mean age of 74.3 ± 7.9 years. Aging is associated with a progressive decline in cardiorespiratory fitness and physiological reserve and is also accompanied by age-related changes in renal function, while cardiovascular and metabolic comorbidities become increasingly prevalent in older populations [42,43]. These factors may contribute to lower exercise capacity and functional performance independently of stroke-related impairment. Although age was not significantly associated with any of the evaluated cardiorespiratory or functional outcomes in the present cohort, this finding should not be interpreted as evidence that aging was clinically irrelevant. The relatively advanced and comparatively restricted age distribution of the cohort may have limited the ability to detect age-related differences, while age may also have indirectly contributed to the observed clinical phenotype through its relationship with comorbidity burden and physiological reserve.

### 4.8. Global and Quantitative Functional Outcome Measures

No significant associations were identified between the examined cardiovascular, metabolic, inflammatory, or lifestyle-related variables and the modified Rankin Scale (mRS). The relatively restricted distribution of mRS scores (median 2, IQR 2–3) may have limited its ability to detect subtle differences in functional status. In contrast, the Barthel Index and 10MWT completion time demonstrated significant associations with several cardiovascular risk factors, suggesting that these measures may be more sensitive than the mRS to functional differences associated with systemic factors in patients with post-stroke spasticity [44,45]. In this population, 10MWT performance may reflect not only systemic cardiorespiratory reserve but also the combined effects of spasticity, weakness, abnormal muscle activation, impaired motor control, and reduced movement efficiency.

### 4.9. Limitations

Several limitations should be considered when interpreting the findings of this study.

First, the retrospective observational design does not permit causal inference between cardiovascular risk factors and the evaluated cardiorespiratory or functional outcomes. Prospective longitudinal studies are needed to determine whether modification of these risk factors is associated with subsequent improvements in rehabilitation outcomes.

Second, this was a single-center study, and the study population may not be fully representative of the broader population of patients with post-stroke spasticity. The relatively advanced mean age of the participants and the high prevalence of cardiovascular and metabolic comorbidities may limit the generalizability of these findings to younger stroke survivors or populations with different cardiovascular risk profiles. Although age was not significantly associated with the evaluated outcomes in the univariate analyses, aging may still have influenced the overall comorbidity burden, renal function, physiological reserve, and functional status of the cohort; therefore, residual age-related confounding cannot be entirely excluded. Furthermore, because the cohort was restricted to patients with ischemic stroke, the present findings cannot be directly generalized to patients with hemorrhagic stroke. In addition, eligibility required the ability to perform CPET, which may have introduced selection bias by favoring patients with sufficient motor function and clinical stability to complete exercise testing. Consequently, patients with more severe motor impairment, greater disability, or comorbidities precluding CPET may have been underrepresented. The findings should therefore be generalized with caution to patients with more severe post-stroke disability or medical comorbidity who are unable to perform CPET. The study also did not include a non-stroke control group matched for age and comorbidity burden. Therefore, it cannot be determined whether the observed associations between cardiovascular, metabolic, and renal factors and cardiorespiratory or functional performance are specific to stroke or post-stroke spasticity, or whether similar relationships would also be observed in older adults without stroke who have comparable comorbidities. Future controlled studies including appropriately matched non-stroke populations are needed to clarify the specificity of these associations.

Third, several potentially relevant clinical variables were unavailable for inclusion in the analytical dataset, including stroke severity, standardized measures of frailty, muscle mass, pre-stroke or baseline functional status, post-stroke depression, lesion volume and imaging characteristics, and pre-stroke physical activity. These factors may influence cardiorespiratory fitness and functional performance after stroke and may therefore have contributed to the observed associations. Although MAS assessment was performed clinically and documented in the medical records, individual MAS scores were not extracted into the analytical dataset. Therefore, the potential contribution of spasticity severity to the observed cardiorespiratory and functional associations could not be evaluated. Consequently, residual confounding related to stroke severity, frailty, muscle mass, baseline functional status, spasticity severity, and other unmeasured clinical factors cannot be excluded despite the multivariable adjustment performed.

Fourth, the 10MWT was based on a single timed trial, reflecting the routine clinical assessment protocol from which the retrospective data were obtained. Because repeated trials were not performed, within-subject variability could not be assessed and a mean value across multiple trials could not be calculated.

Fifth, no formal correction for multiple comparisons was applied because of the exploratory nature of the analyses. Therefore, the reported associations should be interpreted cautiously and confirmed in larger prospective multicenter studies.

### 4.10. Future Directions

The findings of the present study provide several directions for future research. Prospective longitudinal studies are needed to confirm the prognostic value of the cardiovascular, metabolic, inflammatory, and renal factors identified in this cohort and to evaluate their relationship with long-term cardiorespiratory fitness and functional recovery in patients with post-stroke spasticity.

Given the consistent associations observed between eGFR and all evaluated cardiorespiratory and functional outcomes, the role of renal function in post-stroke rehabilitation deserves further investigation. Future studies should clarify the mechanisms underlying these associations and evaluate whether renal function may improve rehabilitation risk stratification.

Interventional studies are also warranted to determine whether optimization of modifiable cardiovascular risk factors, combined with individualized exercise-based rehabilitation, can improve functional outcomes. Growing evidence indicates that structured exercise programs are safe and effective after stroke, while contemporary rehabilitation increasingly incorporates advanced upper-limb interventions and multidisciplinary strategies to maximize functional recovery in patients with post-stroke spasticity [46,47,48]. Finally, large multicenter studies integrating standardized spasticity assessment, advanced neuroimaging, circulating biomarkers, and patient-reported outcome measures are needed to validate these findings and support more individualized rehabilitation strategies.

## 5. Conclusions

This study shows that cardiovascular, metabolic, inflammatory, and renal factors are closely associated with cardiorespiratory fitness and functional performance in patients with post-stroke spasticity. Among the evaluated variables, estimated glomerular filtration rate showed the most consistent independent associations across all assessed cardiorespiratory and functional outcomes, highlighting the potential importance of renal function in rehabilitation assessment.

Poor glycemic control, active smoking, coronary artery disease, systemic inflammation, elevated body mass index, and hypertension were also associated with reduced exercise capacity and functional performance, emphasizing that cardiovascular comorbidity should be considered an integral component of the rehabilitation assessment rather than merely a coexisting clinical condition.

These findings support a comprehensive multidisciplinary rehabilitation approach that combines neurological rehabilitation with systematic cardiovascular risk assessment and optimization of modifiable risk factors. Such an approach may contribute to more individualized rehabilitation strategies and improved functional outcomes.

Overall, systematic evaluation of the cardiovascular risk profile should be incorporated into the routine clinical assessment of patients with post-stroke spasticity to facilitate personalized rehabilitation planning and risk stratification.

## Figures and Tables

**Table 1:** Baseline demographic, clinical, anthropometric, and laboratory characteristics of the study population.

Variable	n (%)	Mean ± SD/Median (IQR)
Sex		
Male	59 (50.4)	
Female	58 (49.6)	
Age (years)		74.30 ± 7.92
BMI (kg/m^2^)		27.79 ± 4.53
Waist circumference (cm)		103.45 ± 15.05
Hypertension	98 (83.8)	
Diabetes mellitus	42 (35.9)	
HbA1c (%)		6.0 (5.7–7.7)
CAD	34 (29.1)	
Dyslipidemia	70 (59.8)	
LDL cholesterol (mg/dL)		102.30 ± 23.78
eGFR (mL/min/1.73 m^2^)		68.33 ± 16.26
Hemoglobin (g/dL)		13.28 ± 1.10
CRP (mg/L)		3.8 (1.95–5.55)
Smoking status		
Never smoker	60 (51.3)	
Former smoker	37 (31.6)	
Current smoker	20 (17.1)	
Stroke location		
Right-sided	64 (54.7)	
Left-sided	49 (41.9)	
Other/Bilateral	4 (3.4)	
Time since stroke (months)		11 (7–20)

Abbreviations: BMI, body mass index; CAD, coronary artery disease; CRP, C-reactive protein; eGFR, estimated glomerular filtration rate;; HbA1c, glycated hemoglobin; IQR, interquartile range; LDL, low-density lipoprotein; SD, standard deviation.

**Table 2:** Multivariable linear regression analysis of factors independently associated with VO_2_peak.

Predictor	B	SE	β	t	*p*-Value	95% CI	VIF
HbA1c	−0.267	0.080	−0.229	−3.324	0.001	−0.426 to −0.108	1.061
eGFR	0.045	0.006	0.508	7.439	<0.001	0.033 to 0.057	1.037
CAD	−0.960	0.216	−0.304	−4.454	<0.001	−1.388 to −0.533	1.038
Current smoking (vs. non-smokers)	−1.131	0.261	−0.297	−4.339	<0.001	−1.647 to −0.614	1.042
CRP	−0.076	0.029	−0.183	−2.634	0.010	−0.134 to −0.019	1.072

**Table 3:** Multivariable linear regression analysis of factors independently associated with anaerobic threshold (AT).

Predictor	B	SE	β	t	*p*-Value	95% CI	VIF
HbA1c	−0.284	0.096	−0.221	−2.959	0.004	−0.474 to −0.094	1.036
eGFR	0.043	0.007	0.441	5.915	<0.001	0.029 to 0.057	1.031
CAD	−0.911	0.258	−0.262	−3.527	0.001	−1.423 to −0.399	1.021
Current smoking (vs. non-smokers)	−1.358	0.312	−0.324	−4.346	<0.001	−1.977 to −0.739	1.027

Abbreviations: AT, anaerobic threshold; B, unstandardized regression coefficient; β, standardized regression coefficient; CAD, coronary artery disease; CI, confidence interval; eGFR, estimated glomerular filtration rate; HbA1c, glycated hemoglobin; SE, standard error; t, t-statistic; VIF, variance inflation factor.

**Table 4:** Multivariable linear regression analysis of factors independently associated with exercise duration.

Predictor	B	SE	β	t	*p*-Value	95% CI	VIF
eGFR	0.020	0.004	0.434	5.458	<0.001	0.013 to 0.027	1.028
Current smoking (vs. non-smokers)	−0.458	0.156	−0.235	−2.940	0.004	−0.768 to −0.149	1.042
CRP	−0.040	0.017	−0.188	−2.334	0.021	−0.074 to −0.006	1.053
Hypertension	−0.322	0.156	−0.162	−2.059	0.042	−0.632 to −0.012	1.005
Diabetes mellitus	−0.175	0.122	−0.114	−1.431	0.155	−0.417 to 0.067	1.040

Abbreviations: B, unstandardized regression coefficient; β, standardized regression coefficient; CI, confidence interval; CRP, C-reactive protein; eGFR, estimated glomerular filtration rate; SE, standard error; t, t-statistic; VIF, variance inflation factor.

**Table 5:** Multivariable linear regression analysis of factors independently associated with maximum workload.

Predictor	B	SE	β	t	*p*-Value	95% CI	VIF
eGFR	0.186	0.038	0.403	4.855	<0.001	0.110 to 0.263	1.022
Current smoking (vs. non-smokers)	−2.377	1.652	−0.120	−1.439	0.153	−5.651 to 0.897	1.022
Diabetes mellitus	−2.075	1.293	−0.133	−1.605	0.111	−4.637 to 0.486	1.016
BMI	−0.371	0.136	−0.224	−2.725	0.007	−0.641 to −0.101	1.000

Abbreviations: B, unstandardized regression coefficient; β, standardized regression coefficient; BMI, body mass index; CI, confidence interval; eGFR, estimated glomerular filtration rate; SE, standard error; t, t-statistic; VIF, variance inflation factor.

**Table 6:** Multivariable linear regression analysis of factors independently associated with 10MWT completion time.

Predictor	B	SE	β	t	*p*-Value	95% CI	VIF
eGFR	−0.033	0.010	−0.278	−3.294	0.001	−0.053 to −0.013	1.016
Current smoking (vs. non-smokers)	1.206	0.438	0.235	2.753	0.007	0.338 to 2.075	1.038
CRP	0.125	0.048	0.222	2.584	0.011	0.029 to 0.220	1.047
CAD	1.080	0.362	0.254	2.985	0.003	0.363 to 1.796	1.029

Abbreviations: B, unstandardized regression coefficient; β, standardized regression coefficient; CI, confidence interval; CRP, C-reactive protein; eGFR, estimated glomerular filtration rate; SE, standard error; t, t-statistic; VIF, variance inflation factor.

**Table 7:** Multivariable linear regression analysis of factors independently associated with Barthel Index score.

Predictor	B	SE	β	t	*p*-Value	95% CI	VIF
eGFR	0.205	0.038	0.408	5.389	<0.001	0.130 to 0.281	1.030
Current smoking (vs. non-smokers)	−7.123	1.640	−0.329	−4.342	<0.001	−10.374 to −3.873	1.033
CAD	−4.808	1.359	−0.268	−3.537	0.001	−7.501 to −2.114	1.032
Diabetes mellitus	−2.627	1.323	−0.155	−1.986	0.050	−5.249 to −0.005	1.091
Dyslipidemia	−3.303	1.281	−0.199	−2.579	0.011	−5.841 to −0.765	1.068

Abbreviations: B, unstandardized regression coefficient; β, standardized regression coefficient; CI, confidence interval; eGFR, estimated glomerular filtration rate; SE, standard error; t, t-statistic; VIF, variance inflation factor.

## Data Availability

The datasets generated and/or analyzed during the current study are available from the corresponding author upon reasonable request. The datasets are not publicly available due to privacy and ethical restrictions.

## Abbreviations

AT	anaerobic threshold
B	unstandardized regression coefficient
β	standardized regression coefficient
BI	Barthel Index
BMI	body mass index
CAD	coronary artery disease
CI	confidence intervals
CPET	cardiopulmonary exercise testing
CRP	C-reactive protein
eGFR	estimated glomerular filtration rate
Hb	hemoglobin
HbA1c	glycated hemoglobin
IQR	interquartile range
LDL	low-density lipoprotein
MAS	Modified Ashworth Scale
mRS	Modified Rankin Scale
R^2^	coefficient of determination
PSS	post-stroke spasticity
SE	standard error
SD	standard deviation
VE	ventilatory equivalents
VO_2_peak	peak oxygen uptake
VCO_2_	carbon dioxide production
VIF	variance inflation factor
VO_2_	oxygen uptake
10MWT	10-Meter Walk Test
